# Operative Risk Factors and Microbiologic Profiles of Deep Infection Following Pilon Fracture Fixation

**DOI:** 10.3390/microorganisms13122837

**Published:** 2025-12-13

**Authors:** Jackson M. Cathey, Crystal Jing, Julia E. Ralph, Kathleen Chang, Alexandra Krez, Joshua Helmkamp, Anna Bryniarski, Conor O’Neill, Samuel Adams, Albert T. Anastasio

**Affiliations:** 1 School of Medicine, Duke University Health System, Duke University, 40 Duke Medicine Circle, Durham, NC 27710, USA; jackson.cathey@duke.edu (J.M.C.);; 2 Department of Orthopaedic Surgery, Duke University Health System, Duke University, 2301 Erwin Road, Durham, NC 27710, USA

**Keywords:** fixation, fracture, infection, operative time, ORIF, pilon

## Abstract

Deep tissue infection is a serious complication following operative fixation of pilon fractures, yet the influence of intraoperative factors and microbial characteristics remains underexplored. In this retrospective review of 123 patients treated with open reduction and internal fixation, we evaluated demographic, injury-related, operative, and microbiologic variables associated with deep infection. Patients who developed infection had longer operative times than those who did not. This modestly powered study found that operative duration was not independently associated with infection risk after adjustment for key covariates. Larger studies are needed to confirm this finding. Deep infection rates did not differ by surgical approach or time to fixation. Nine patients (7.3%) developed deep infection, with cultures demonstrating a heterogeneous microbial profile most commonly including *Pseudomonas aeruginosa*, *Enterococcus faecalis*, *Staphylococcus aureus*, and *Enterobacter cloacae*. Deep infections resulted in nonunion in 67% (6/9), PTOA in 44% (4/9), and amputation in 33% (3/9) of cases.

## 1. Introduction

Fractures of the distal tibial plafond, or pilon fractures, are complex injuries that pose significant challenges in management [1]. Pilon fractures account for approximately 1 in every 6 fractures of the tibia, and less than 1% of all fractures [2]. Frequently resulting from high-energy mechanisms such as motor vehicle accidents or falls from height, pilon fractures are often associated with severe comminution and extensive soft tissue damage [3]. Although rare, these fractures can have devastating long-term impacts on functional outcome and quality of life, with significant morbidity in the short term [3]. Due to the thin tissue envelope around the ankle and high incidence of open injuries, pilon fractures carry a particularly elevated risk of infection [4]. Following operative management, the risk of deep tissue infection is reported to be as high as 20% for open, complete-articular fracture patterns [5,6,7].

Prior studies have examined the risk factors for infection following operatively managed pilon fractures [6,8,9,10]. Patient-specific factors (e.g., increased age, diabetes, active smoking) and injury characteristics (e.g., open fractures, highly comminuted fractures) have consistently been predictive of postoperative infection [5,6,8,9,10,11,12]. Operative risk factors for deep infection remain poorly characterized. Operative treatment with reduction and internal fixation (ORIF) remains the gold standard for fixation [13]. However, there exists a substantial degree of procedural heterogeneity, as decisions regarding surgical approach and hardware configuration vary by provider and fracture pattern [14]. The impact of these surgical decisions on infection rates is not well studied, likely reflecting an emphasis on fracture alignment, with infection risk being a secondary consideration. There also remains a paucity of literature describing the microbiologic spectrum of these infections. Deep surgical site infections (SSIs) can arise from a range of sources (e.g., intraoperative contamination, postoperative wound breakdown, hematogenous seeding) and may involve diverse organisms with distinct prognostic indications [15].

Deep infection is a particularly strong predictor of poor outcomes following pilon fracture, including nonunion, post-traumatic osteoarthritis (PTOA), and in severe cases, amputation due to persistent or uncontrolled infection [8,16]. Given the high baseline risk of infection following pilon fracture, it is important to identify modifiable risk factors for deep infection and better understand the microbiota driving adverse clinical sequelae. By examining a series of patients with pilon fractures managed definitively via ORIF, we aimed to (1) elucidate any operative characteristics that might influence deep infection rate and (2) report our institutional experience with deep SSI management and the associated microbiologic profiles of these infections. We hypothesized that prolonged operative time would be predictive of deep tissue infection. Secondarily, we explored the microbial profile of deep SSIs and their clinical consequences.

## 2. Materials and Methods

This study protocol was examined by our Institutional Review Board and deemed exempt from full committee review. This study adhered to STROBE (Strengthening the Reporting of Observational Studies in Epidemiology) guidelines [17].

**Figure 1:**
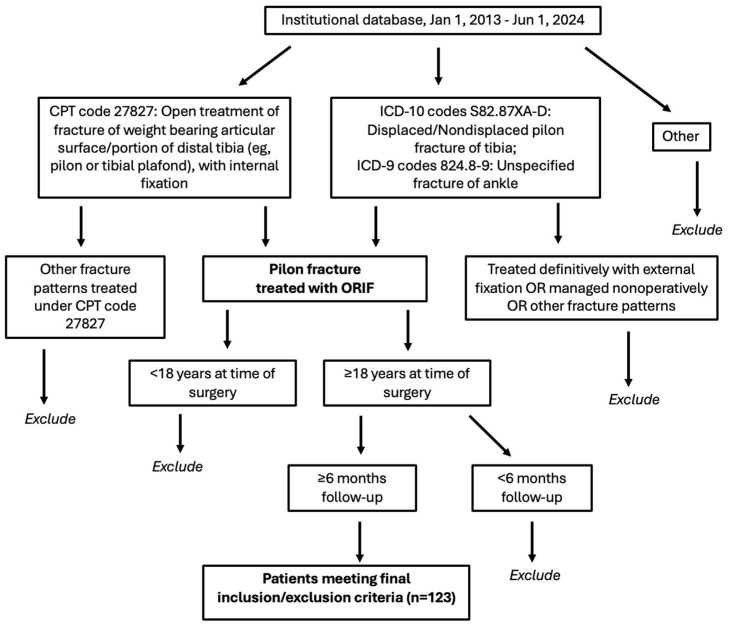
Patient inclusion/exclusion criteria flowchart. CPT: current procedural terminology; ICD: international classification of diseases; ORIF: open reduction and internal fixation.

We retrospectively reviewed all patients ≥ 18 years of age who underwent surgical fixation of pilon fractures between 1 January 2013 and 1 June 2023, at a single, large academic institution. Patients were identified using Current Procedural Terminology (CPT) code 27827 and cross-referenced with International Classification of Diseases, Ninth and Tenth Revision (ICD-9, 10) codes 824.8-9 and S82.87XA-D to minimize missed cases. Patient charts were reviewed to select only those with pilon fractures treated definitively via ORIF who had at least 6 months of follow-up (Figure 1).

Perioperative protocols remained constant over the 10-year analysis period. Initial management consisted of closed reduction and splinting, followed by irrigation and debridement for any open fractures within 24 h of presentation to the emergency department (ED). Select cases were treated via a staged approach, with external fixation within 36 h of ED presentation, followed by delayed definitive fixation. Definitive fixation was only performed when the degree of swelling was amenable to operation, such that definitive closure could be performed without undue skin tension. Once soft tissues were deemed optimized, patients underwent ORIF with locking plates and/or percutaneous intramedullary nailing (IMN) and intra-articular reduction. Adjunctive fibular nailing was performed when distal fractures threatened stability or there was concern that comminution might affect tibial alignment. The surgical approach was based on the surgeon’s preference and injury pattern. All patients were kept non-weight bearing for the first 6 weeks; progression to partial- and full-weight bearing was subject to provider protocol and clinical progression.

Data was collected via manual chart review. Demographic characteristics included age, sex, smoking status, body mass index (BMI), and history of diabetes mellitus. Injury characteristics included open versus closed fracture, incidence of polytrauma, and AO/OTA (Arbeitsgemeinschaft für Osteosynthesefragen/Orthopaedic Trauma Association) fracture classification. Preoperative radiographs were evaluated by a single rater to determine AO/OTA class. Operative characteristics included time from consult to definitive fixation (days), staged versus immediate ORIF, surgical approach, hardware configuration (locking plates alone, IMN alone, dual construct with IMN and locking plates), and operative time (minutes). Post-surgical deep infection was defined as infection of the deep soft tissues underlying the surgical site, within 90 days following ORIF [18]. Cultures were collected for all patients with deep infection at the time of irrigation and debridement. Collection of Gram stain and multiple aerobic and anaerobic cultures from the wound site was standard. Cultures were monitored for 5 days for growth. Adverse clinical outcomes included nonunion, progression to amputation, development of PTOA by final follow-up, and progression to fusion or total ankle arthroplasty (TAA). Nonunion was determined by reviewing postoperative radiographs. Nonunion was defined as persistent fracture lucency on radiographs, or <50% osseous bridging on computed tomography, without interval healing at ≥9 months postoperatively [19,20]. Postoperative radiographs were also utilized to determine PTOA, defined by a Kellgren-Lawrence score of 2 or higher by final follow-up [21].

Demographic data was reported as means ± standard deviations (SDs) or proportions and percentages. Patients were stratified by presence of deep infection. Bivariate analyses were performed to compare operative characteristics between patients with and without deep infection. Chi-square omnibus tests and Fisher’s exact tests were used for categorical variables (approach, hardware, staged ORIF); effect sizes were measured using Cramer’s V. Wilcoxon rank-sum tests were used for continuous variables (operative time, time to fixation); effect sizes were measured using rank-based effect size *r*. To evaluate whether operative characteristics were independently predictive of deep infection, a multivariable regression analysis was performed. This parsimonious model included operative characteristics with *p*-values < 0.10 and patient- and injury-specific risk factors for deep tissue infection following pilon fracture fixation. Selection of operative characteristics was iterative, while patient/injury-level factors were defined a priori [5]. A z-score transformation was applied to continuous variables to allow for standardized comparisons between covariates. All patients had data for each covariate of interest. The model, therefore, reflected a complete case analysis. Results were presented as odds ratios (ORs) with 95% confidence intervals (CIs). All significance testing was 2-tailed with a significance threshold of *p* < 0.05. Post hoc power analysis for a sample size of 123 patients and α = 0.05 revealed 44% power to detect a difference in the a priori primary outcome measure (operative time) between patients with and without deep infection. Findings should therefore be interpreted in the context of relative effect sizes. All statistical analyses were completed in MATLAB (Version R2024b. Natick, MA, USA).

## 3. Results

### 3.1. Demographic, Injury, and Operative Characteristics

**Figure 2:**
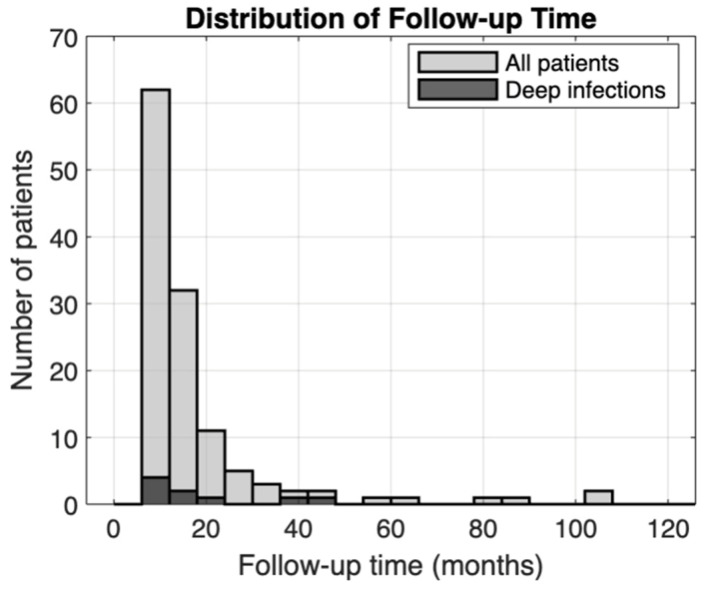
Follow-up time distribution. Histogram bins correspond to 6-month time periods.

A total of 123 patients with an average follow-up of 17.2 ± 17.5 months (median = 11.8 months, interquartile range = 7.7–17.6 months, range = 6.0–107 months) were included (Figure 2). Patients were predominantly male (n = 67, 54.5%) with a mean age of 46.3 ± 15.9 years and a mean BMI of 30.0 ± 7.1. Twelve patients had a history of diabetes (9.8%), while 17 (13.8%) were active smokers at the time of injury. A substantial proportion of patients had polytraumatic injuries (n = 47, 38.2%) and open pilon fractures (n = 34, 27.6%). The majority of patients had complete articular fractures (n = 78, 63.4%); 37 patients had partial articular fractures (30.1%), while just 8 patients had extra-articular fractures (6.5%). The average time from consultation to definitive fixation with hardware placement was 12.0 ± 18 days. Most patients were treated via a staged approach, with external fixation followed by ORIF (n = 77, 62.6%). The most common surgical treatment was fixation with a locking plate alone (n = 96, 78.0%), followed by dual construct (n = 15, 12.2%), and IMN (n = 12, 9.8%). The most common surgical approach was direct anterior (n = 36, 29.2%), followed by anterolateral (n = 26, 21.1%), anteromedial (n = 18, 14.6%), lateral (n = 15, 12.2%), medial (n = 10, 8.1%), posterolateral (n = 10, 8.1%), and posteromedial (n = 8, 6.5%). Mean operative time was 192 ± 106 min.

### 3.2. Influence of Operative Factors on Deep Tissue Infection

Among these 123 patients, 14 (11.4%) developed postoperative SSI. Nine of these were deep tissue infections (7.3%) while 5 were superficial (4.1%). Patients with deep infection had an average follow-up of 19.0 ± 14.0 months (median = 17.0 months, interquartile range = 8.5–24.5 months, range = 6.8–45.0 months). Bivariate analyses revealed that patients who developed postoperative deep tissue infection had longer operative times than those who did not (274 ± 122 min vs. 185 ± 102 min, *p* = 0.007) (Table 1). There were no significant differences in the distribution of surgical approach between patients with and without deep infection. The most common surgical approach in the deep infection cohort was an anterolateral approach (3/9), while a direct anterior approach was the most common in patients without deep infection (32/114). Time to definitive fixation was similar between patients with deep infection (10.8 ± 6.7 days) and without deep infection (12.1 ± 18.6 days). A similar proportion of patients in each cohort underwent staged ORIF. Hardware configuration was likewise not found to be a significant predictor of deep infection in this sample. Although not reaching statistical significance, possibly due to small sample size, no infections occurred among 12 IMN cases versus 20% (3/15) in dual construct cases, warranting further investigation.

On multivariable analysis, operative time was not found to be a significant predictor of deep infection in this sample (Table 2). Though all included operative and patient/injury-specific covariates were associated with an OR > 1.0, none reached statistical significance. The strongest predictors of deep infection were diabetes mellitus (OR = 3.56; 95% CI [0.50–25.2], *p* = 0.20) and dual construct fixation (OR = 2.93; 95% CI [0.53–16.4], *p* = 0.22).

### 3.3. Microbial Profile of Deep Tissue Infections and Clinical Outcomes

Intraoperative cultures were collected for the 9 patients who developed deep SSI following pilon fracture fixation (Table 3). Infection diagnoses occurred at an average of 83.0 ± 84.6 days following ORIF (median = 60 days, interquartile range = 17–93 days, range = 5–266 days). The most common causal pathogens were *Pseudomonas aeruginosa* (n = 3), *Staphylococcus aureus* (overall: n = 3; MRSA: n = 2; MSSA: n = 1), *Enterococcus faecalis* (n = 2), and *Enterobacter cloacae* (n = 2). Five patients developed polymicrobial infections (5/9). Most patients required multiple operative wound irrigations (6/9) (Table 4). Two patients with soft tissue infection but no evidence of osteomyelitis were successfully treated with hardware retention and oral antibiotic regimens (2/9). The remaining patients underwent hardware removal and treatment with at least 6 weeks of intravenous antibiotics, often followed by an oral regimen narrowed to the spectrum of microbiota collected at final operative wound irrigation (7/9). Eight patients had adverse clinical sequelae following deep infection (8/9). The most common adverse clinical outcomes were nonunion (n = 6), PTOA (n = 4), and amputation (n = 3). Only two patients with deep infections achieved osseous union by final follow-up (2/9); union status could not be evaluated in one patient who underwent BKA in the acute postoperative setting. An additional 5 patients developed superficial SSI; 2 were pin site infections, while the other 3 were infections around the wound site. Cultures were collected for 1 patient with superficial SSI and resulted as positive for *Pseudomonas aeruginosa* and MSSA. This patient recovered after antibiotic therapy and 2 operative wound irrigations. The remaining patients with superficial SSIs recovered with antibiotic therapy alone (4/5). Among these 5 cases, no patients developed nonunion or required amputation or TAA/fusion (0/5); however, 2 developed PTOA by final follow-up (2/5).

## 4. Discussion

Deep tissue infection remains one of the most feared complications following operative fixation of pilon fractures. While prior studies have identified several patient- and injury-level risk factors, few have examined how operative decisions influence the risk of deep infection. Similarly, there remain few reports of the specific microbes driving postoperative deep infection. In this modestly sized single-center series of patients with pilon fractures treated with ORIF, we sought to determine which operative variables modulate deep infection risk. Deep infections occurred in nine individuals (7.3%). Lengthened operative time showed a strong association with deep infection on bivariate analysis but was not independently predictive after adjustment. However, with only 44% power to detect such associations, these null findings should not be interpreted as definitive evidence against an operative time effect. Deep infection rates were similar across surgical approaches and staged versus immediate ORIF. Earlier fixation was not associated with increased risk. Overall, the deep infections in our study cohort were associated with a wide range of causal pathogens, most often polymicrobial. These infections posed significant challenges in clinical management and commonly resulted in adverse sequelae, including nonunion, PTOA, and amputation.

Lengthened operative time is associated with an increased risk for SSI across various procedures [22]. Several studies have reported increased odds of infection with longer operative times in the context of pilon fracture fixation. A recent meta-analysis of ten retrospective pilon fracture management studies found longer surgical duration to be predictive of overall SSI occurrence [6]. In a series of 175 patients with AO/OTA 43C pilon fractures, one study found increased operative time to be predictive of infections requiring irrigation and debridement [23]. Authors also found a positive relationship between SSI and risk and number of plates used for fixation. We similarly observed a higher incidence of infection among patients treated with dual constructs and no infections among those treated with tibial nailing alone, though this result was not significant in our limited sample. Although prior work has implicated operative factors in the development of deep infection after pilon fracture fixation, isolating the independent effects of operative time and hardware configuration remains challenging because both tend to increase with greater fracture complexity. Our cohort included a range of fracture complexities and varying degrees of soft tissue compromise. In this context, our adjusted analyses did not identify these variables as independently predictive of infection, a result that may reflect limited statistical power rather than a true absence of association. Differences in institutional protocols, including standardized antibiotic prophylaxis and re-dosing practices, may also attenuate time-related infection risks reported in earlier studies. Additionally, the relatively low infection rate in our cohort suggests stronger baseline infection control, potentially reducing the measurable impact of operative duration. These considerations underscore the need for caution in interpreting our findings. While fracture reduction quality should remain the operative priority, surgeons should continue to minimize unnecessary operative time as a prudent infection-prevention measure. Larger studies are required to better define operative time thresholds associated with increased risk.

Our study did not identify surgical approach to be predictive of deep infection risk. Likewise, a recent multicenter, retrospective review of 581 patients with pilon fractures treated via ORIF found no differences between surgical approach and risk of deep infection [24]. The majority of surgeries were performed via a medial approach. Authors concluded that approach selection need not account for infection risk, encouraging surgeons to select the approach that best addresses the specific fracture pattern. Accounting for the influence of fracture pattern, a later study explored the influence of surgical approach on deep infection risk within a population of 150 patients with 43C type fractures [5]. Authors noted an increased risk of deep infection with the posterolateral approach. Posterolateral approaches were infrequently utilized at our institution. The low infection rate with the posterolateral approach (1/10) in our series should not be over-interpreted, given the small sample size and wide confidence intervals. It also remains unknown if fracture patterns in our cohort influenced results via the posterolateral approach. Yet given the largely similar distribution of infection risk across the seven included surgical approaches, our findings align with the recommendations of Esposito et al. [24]; surgical approach does not appear to have any independent influence on risk for deep infection. For closed fractures, these results support the notion that surgeons may select their operative approach based on familiarity and preoperative injury evaluation. Options may be more limited for open fractures, in which surgical approach is often dictated by soft tissue condition and location of the open wound. Though it is generally recommended to minimize vascular compromise [7], our findings do not indicate that any particular approach needs to be avoided in the aim of mitigating deep infection risk.

We did not note any significant differences in time to definitive fixation between cohorts in our modestly sized series. A recent study comparing early versus delayed fixation of pilon fractures found that delayed surgery was associated with a reduced risk for infection [25]. Conversely, authors found that early surgery was associated with shorter healing time and stronger functional outcome, but advised caution with early fixation due to this risk. Similar studies have not found any difference in infection rates between early and delayed fixation [26,27]. In our sample, mean times to definitive fixation exceeded the threshold for early fixation [27]. Thus, while our results did not indicate that time to fixation was a key modulator of deep infection risk, the effect of early fixation remains unclear. This is likely driven by our institutional protocol, wherein surgeons delayed definitive fixation until the degree of soft tissue swelling was amenable to operation.

Our patient series exhibited a deep infection rate of 7%. Deep infection rates in other series of operatively managed pilon fractures have ranged from 10% to 20%, with higher rates observed for open and complex fractures [5,8,26]. This discrepancy might be explained by our inclusion of closed fractures and less complex fracture patterns. The high proportion of immediate ORIF (37%) might have also played a role in the observed infection rate, as deep infection rates following primary ORIF have been reported to be between 2% and 6% [3,28]. The most common causal pathogens of deep infection in our study were *Pseudomonas aeruginosa*, *Enterococcus faecalis,* MRSA, and *Enterobacter cloacae*. This microbiological spectrum is similar to that reported by Molina et al., who studied a series of 52 patients with pilon fracture who developed deep SSI postoperatively [29]. Authors found that *Enterobacter* species contributed to 19% of infections (14/52). However, the most commonly noted microbe was MSSA; several similar studies have reported comparable proportions of MSSA growth in the context of postoperative pilon fixation infection [8,30]. Though we only noted one case of MSSA deep infection, it remains plausible that this rate might have been higher in a larger cohort. The relative predominance of Gram-negative and enterococcal isolates in our series may reflect the high burden of open injuries, soft tissue compromise, and staged fixation, which together increase exposure to nosocomial and polymicrobial contamination [31]. These patterns likely mirror our institutional flora and perioperative antibiotic practices and should be interpreted in the context of local antibiograms when extrapolating empiric coverage recommendations.

Deep infections following pilon fracture fixation often carry a poor prognosis [32]. Similar to prior studies, we noted a remarkably high rate of nonunion and PTOA among patients with deep SSI [8]. The high rate of treatment failure in our series (6/9 nonunion) likely reflects biofilm formation on hardware, which protects bacteria from both antibiotics and host immunity, often necessitating hardware removal for infection eradication. *Pseudomonas* and *Enterococcus* are particularly adept biofilm-formers [33]. All patients with such isolates were treated with hardware removal. Perhaps most notably, three patients with deep infection necessitated amputations to preserve the leg proximal to the site of ongoing infection. Two of the patients who underwent amputations had polymicrobial infections with isolates known to form biofilms. The third patient had a persistent infection with *Enterococcus faecalis*. Albeit a very limited and potentially biased patient series, these culture findings align with the overarching body of evidence supporting the utility of microbial profiles as prognostic indicators. In the setting of diabetic foot ulcers, polymicrobial infections have been demonstrated to carry a higher risk of need for amputation than monomicrobial infections [34,35]. Following pilon fracture fixation, amputation risk has not been specifically explored, but polymicrobial infection has been predictive of adverse clinical outcomes at large [8]. While prior work has not explored the relationship of *Enterococci* and amputation risk, *Enterococci* are often the central node of fracture-related infections, frequently co-occurring with other high-risk pathogens such as *Pseudomonas* and *Enterobacter* [36]. Our findings add to the evolving evidence of polymicrobial infections as poor prognostic indicators. Source control is central to management, as these patients often require repeated debridement, hardware removal, and flap coverage. Early, coordinated surgical and infectious-disease care is essential in cases of polymicrobial deep infection.

This study has several limitations. This institutional series was entirely retrospective, limiting the accuracy of data to that available in electronic health records. We could not control for miscoded data, which might have led to the missed detection of cases that would have otherwise fit our inclusion criterion. Though the overarching management of pilon fractures was standardized at our institution, not all patients were subject to equivalent clinical monitoring. This limitation was particularly accentuated in our reporting of adverse outcomes, as nonunion and PTOA were both determined from radiographs, introducing the potential for missed detection of these diagnoses when imaging was not collected at final follow-up. This patient series was also limited in sample size. Though our institution handles a relatively large volume of pilon fractures, the overall incidence of deep infection was rare. As such, this study may be underpowered to detect certain operative risk factors for deep SSI that could have been uncovered with a larger cohort. Though our study detected a significant difference in operative time between cohorts on bivariate analysis, multivariable analyses were likely underpowered. This limited sample of deep infection cases precluded any statistical analyses of microbiologic predictors of clinical prognosis. These results should thus be interpreted cautiously; our findings reflect those of a single institutional experience rather than generalizable findings. As the spectrum of microbiota varies by region, this bias is likely only to be exacerbated in a study of such a small sample size. Similarly, the heterogeneity within our cohort limits the generalizability of our findings. This patient series comprised a range of AO/OTA class injuries, and though we selected for only those treated definitively via ORIF, it is possible that prior external fixation, operator-dependent variables, and patient demographics might have contributed to deep infection risk more so than any operative variable alone. It remains difficult to estimate the role of surgical approach retrospectively, as a range of surgeons performed each approach, introducing procedural heterogeneity within and between approach subgroups. A larger study with an a priori power analysis would be integral in further evaluating operative predictors of infection risk. In studying the role of surgical approach in deep infection risk, a prospective design would be best-suited, as the present study cannot elucidate the independent effect of approach, given the observed variance in patient and injury characteristics, as well as the inclusion of multiple operating surgeons.

Nonetheless, our findings indicate that operative variables such as surgical approach, time to fixation, and hardware configuration were not independently predictive of deep infection following pilon fracture fixation. Deep infections, although relatively uncommon in this patient series, were associated with diverse and often polymicrobial pathogens and carried a high risk of adverse outcomes, including nonunion, PTOA, and amputation. These results emphasize the need for ongoing efforts to mitigate perioperative infection risk and for future studies to better identify microbial and host factors driving poor prognoses in this high-risk population. Prospective, multicenter studies will be integral in elucidating operative risk factors for infection.

## 5. Conclusions

In this institutional series of 123 pilon fractures managed with ORIF, deep infection was infrequent but carried substantial morbidity. Patients who developed infection had longer operative times on bivariate analysis; however, though operative time showed a strong bivariate association with infection (*p* = 0.007), it was not independently predictive after adjustment in our modestly powered analysis (44% power). Larger multicenter studies are needed to definitively establish whether operative time is a modifiable risk factor. Similarly, surgical approach, time to fixation, and hardware configuration were not associated with increased infection risk, suggesting that patient and soft tissue factors may exert greater influence than intraoperative choices alone. The microbial spectrum of deep infections was heterogeneous and often polymicrobial, most commonly involving *Pseudomonas aeruginosa*, *Enterococcus faecalis*, and *Enterobacter cloacae*, and was associated with high rates of nonunion, PTOA, and limb loss. These findings underscore the importance of early recognition, aggressive source control, and culture-directed therapy. Future work should focus on large, prospectively collected, multicenter datasets that can (1) define operative time and hardware-related thresholds for infection within specific AO/OTA fracture classes and soft tissue grades and (2) clarify how polymicrobial and biofilm-forming organisms influence nonunion, PTOA, and amputation risk, thereby informing targeted prophylactic and treatment strategies tailored to local antibiograms.

## Figures and Tables

**Table 1:** Operative characteristics of surgically managed pilon fractures among patients with and without deep infection.

Covariate	Deep Infection (n = 9)	No Deep Infection (n = 114)	*p*-Value	Effect Size
**Hardware**			0.097	Cramer’s V = 0.20
Locking plate alone	6 (66.7%)	90 (78.9%)		
IMN	0 (0.0%)	12 (10.5%)		
Dual construct	3 (33.3%)	12 (10.5%)		
**Approach**			0.67	Cramer’s V = 0.18
Anterolateral	3 (33.3%)	25 (21.9%)		
Anteromedial	0 (0.0%)	18 (15.8%)		
Direct anterior	2 (22.2%)	32 (28.1%)		
Posterolateral	1 (11.1%)	9 (7.9%)		
Posteromedial	1 (11.1%)	7 (6.1%)		
Lateral	2 (22.2%)	13 (11.4%)		
Medial	0 (0.0%)	10 (8.8%)		
**Operative time (minutes)**	274 ± 123	185 ± 102	**0.007**	r = 0.24
**Staged ORIF**	70 (61.4%)	7 (77.8%)	0.048	Cramer’s V = 0.09
**Time to definitive** **fixation (days)**	10.8 ± 6.7	12.1 ± 18.6	0.48	r = 0.06

IMN: intramedullary nailing; ORIF: open reduction and internal fixation. Values are presented as n (%) or mean ± standard deviation. Bolded *p*-values represent statistical significance at *p* < 0.05.

**Table 2:** Multivariable analysis of risk factors for deep infection following pilon fracture fixation.

Covariate	OR (95% CI)	*p*-Value
Dual construct	2.93 (0.53–16.4)	0.22
Operative time *	1.51 (0.81–2.83)	0.20
Diabetes mellitus	3.56 (0.50–25.2)	0.20
Open fracture	2.01 (0.40–10.1)	0.40
AO/OTA 43C3	2.33 (0.46–11.8)	0.31

OR: odds ratio; CI: confidence interval; AO/OTA: Arbeitsgemeinschaft für Osteosynthesefragen/Orthopaedic Trauma Association. * z-score transformation.

**Table 3:** Deep infection patient series: patient, operative, and microbial characteristics.

Patient ID	Age (Years)	Sex	Fracture Pattern (AO/OTA)	Operative Technique	Time from Surgery to Infection Diagnosis (Days)	Organism	Polymicrobial
**1**	33	M	43C3.2, closed	Locking plate (staged ORIF), direct anterior approach	17	*MSSA*	No
**2**	79	F	43A3.3, closed	Locking plate (immediate ORIF), posteromedial approach	168	*Pseudomonas aeruginosa*	No
**3**	57	M	43C3.3, closed	Locking plate (staged ORIF), anterolateral approach	63	*Enterococcus faecalis*, *Peptoniphilus asaccharolyticus*, *Proteus mirabilis*, *Pseudomonas aeruginosa*, *Streptococcus agalactiae Group B.*	Yes
**4**	25	M	43C3.1, closed	Locking plate (staged ORIF), direct anterior approach	93	*MRSA*, *Eikenella corrodens*, *Streptococcus constellatus*, *Pseudomonas aeruginosa*, Mixed aerobic and anaerobic organisms,	Yes
**5**	58	M	43C2.1, open	Dual construct (staged ORIF), anterolateral approach	266	*MRSA*, *Streptococcus dysgalactiae group C, Streptococcus mitis group (viridans Streptococcus)*	Yes
**6**	35	M	43C3.2, open	Locking plate (staged ORIF), lateral approach	59	*Enterococcus faecalis*	No
**7**	54	M	43A3.2, open	Dual construct (staged ORIF), lateral approach	5	*Enterobacter aerogenes*, *Enterobacter cloacae*, *Enterococcus (ampicillin susceptible)*, *Klebsiella oxytoca*	Yes
**8**	20	M	43C3.3, open	Dual construct (staged ORIF), anterolateral approach	16	*Enterobacter cloacae*	No
**9**	74	F	43B3.2, closed	Locking plate (immediate ORIF), posterolateral approach	60	Mixed aerobic and anaerobic organisms	Yes

AO/OTA: Arbeitsgemeinschaft für Osteosynthesefragen/Orthopaedic Trauma Association; ORIF: open reduction and internal fixation; MSSA: methicillin-susceptible *staphylococcus aureus*; MRSA: methicillin-resistant *staphylococcus aureus*.

**Table 4:** Deep infection patient series: treatment and clinical outcomes.

Patient ID	Antibiotic Regimen	No. of OR Washouts	Hardware Management & Additional Operations	Union Status	PTOA	Fusion/TAA	Amputation	Final Status & Follow-Up Duration
**1**	PO doxycycline and PO ciprofloxacin x6 months	4	Coverage with radial forearm free flap at 1-month post-ORIF; hardware retention	Nonunion	No	No	No	Full weightbearing with viable flap coverage and no recurrence of infection at 10 months follow-up
**2**	IV cefepime x6 weeks	1	Fibular osteotomy at 5 months post-ORIF; hardware retention	Nonunion	Yes, at 61 weeks post-ORIF	No	No	Full weightbearing, persistent valgus deformity and hindfoot and lower extremity shortening, no recurrence of infection, and asymptomatic PTOA, at 17 months follow-up
**3**	IV piperacillin/tazobactam x6 weeks	2	Placement of external fixator, plate removal, and coverage with proximal thigh STSG at 2 months post-ORIF for infected nonunion with hardware failure; external fixator removed 3 months after placement; repair of nonunion at 7 months post-ORIF with 3 weeks in hexapod external fixator	Nonunion	Yes, at 30 weeks post-ORIF	No	No	Full weightbearing, no recurrence of infection, and asymptomatic PTOA at 17 months follow-up
**4**	IV vancomycin, IV ceftriaxone, and PO metronidazole x6 weeks, followed by PO doxycycline x6 weeks and PO amoxicillin/clavulanate x36 weeks	1	Plate removal and radial forearm free flap coverage at 16 weeks post-ORIF	Nonunion	Yes, at 50 weeks post-ORIF	Yes, arthrodesis at 117 weeks post-ORIF 2/2 severe PTOA	No	Full weightbearing, no recurrence of infection, and symptomatic PTOA at 45 months follow-up
**5**	IV vancomycin x6 weeks	3	Dual construct removal and coverage with medial gastrocnemius flap and STSG from posterior thigh at 2 months post-ORIF	Union by 37 weeks post-ORIF	Yes, at 31 weeks post-ORIF	No	No	Partial weightbearing (in setting of polytrauma, concomitant ipsilateral femur fracture) and symptomatic PTOA at 9 months follow-up
**6**	IV daptomycin x6 weeks, followed by PO doxycycline x6 weeks and PO amoxicillin x8 months (until BKA)	1	Placement of antibiotic nail, plate removal, and coverage with anterolateral thigh free flap at 2 months post-ORIF	Nonunion	No	No	Yes, BKA at 8 months post-ORIF 2/2 ongoing deep tissue infection with concurrent osteomyelitis	Ambulatory with prosthesis at 20 months follow-up without infection recurrence
**7**	IV vancomycin and IV piperacillin/tazobactam x6 weeks, PO ciprofloxacin x8 weeks	3	Vastus lateralis flap coverage at time of attempted limb salvage and ORIF; dual construct removal at time of BKA (5 days post-ORIF)	NA (acute BKA)	NA (acute BKA)	NA (acute BKA)	Yes, BKA 2/2 at 5 days post-ORIF deep tissue infection (contaminated hardware) and failed flap; conversion AKA 2 weeks thereafter 2/2 insufficient source control	Ambulatory with prosthesis 8 months follow-up without infection recurrence
**8**	PO ciprofloxacin and PO doxycycline x6 weeks, followed by PO trimethoprim/sulfamethoxazole x6 weeks	3	Hardware retention	Union by 10 weeks post-ORIF	No	No	No	Full weightbearing at 7 months follow-up without recurrence of infection
**9**	IV vancomycin x6 weeks and PO ciprofloxacin, recurrent courses over 15 months (until BKA)	5	Plate removal at 2 months post-ORIF	Nonunion	No	No	Yes, BKA at 15 months post-ORIF 2/2 recurrent diabetic foot ulcer	Non ambulatory at 39 months follow-up; underwent contralateral BKA 2/2 diabetic foot ulcer; no ipsilateral infection recurrence

No.: number; PTOA: post-traumatic osteoarthritis; TAA: total ankle arthroplasty; PO: per os: ORIF: open reduction and internal fixation; IV: intravenous; STSG: split-thickness skin graft; 2/2: due to; BKA: below-knee amputation; AKA: above-knee amputation.

## Data Availability

The data presented in this study are available on request from the corresponding author as the data underlying this study contain protected health information and cannot be shared publicly due to institutional and ethical restrictions. De-identified data may be made available from the corresponding author upon reasonable request and with appropriate institutional approvals.

## Abbreviations

The following abbreviations are used in this manuscript:

AO/OTA	Arbeitsgemeinschaft für Osteosynthesefragen/Orthopaedic Trauma Association
BKA	Below-Knee Amputation
BMI	Body Mass Index
CI	Confidence Interval
CPT	Current Procedural Terminology
ED	Emergency Department
IMN	Intramedullary Nail
ICD	International Classification of Diseases
IRB	Institutional Review Board
MSSA	Methicillin-Susceptible *Staphylococcus aureus*
MRSA	Methicillin-Resistant *Staphylococcus aureus*
OR	Odds Ratio
ORIF	Open Reduction and Internal Fixation
PTOA	Post-Traumatic Osteoarthritis
SD	Standard Deviation
SSI	Surgical Site Infection
STROBE	Strengthening the Reporting of Observational Studies in Epidemiology
TAA	Total Ankle Arthroplasty

## References

[1] Murawski C.D., Mittwede P.N., Wawrose R.A., Belayneh R., Tarkin I.S. (2023). Management of High-Energy Tibial Pilon Fractures. J. Bone Jt. Surg. Am..

[2] Wennergren D., Bergdahl C., Ekelund J., Juto H., Sundfeldt M., Möller M. (2018). Epidemiology and incidence of tibia fractures in the Swedish Fracture Register. Injury.

[3] Helfet D.L., Koval K., Pappas J., Sanders R.W., DiPasquale T. (1994). Intraarticular “pilon” fracture of the tibia. Clin. Orthop. Relat. Res..

[4] Kottmeier S.A., Madison R.D., Divaris N. (2018). Pilon Fracture: Preventing Complications. J. Am. Acad. Orthop. Surg..

[5] Spitler C.A., Hulick R.M., Weldy J., Howell K., Bergin P.F., Graves M.L. (2020). What are the Risk Factors for Deep Infection in OTA/AO 43C Pilon Fractures?. J. Orthop. Trauma.

[6] Li P.Z., He J.W., Xia X.S. (2025). Risk factors for surgical site infections in patients with pilon fractures: A systematic review and meta-analysis. J. Foot Ankle Surg..

[7] Olson J.J., Anand K., Esposito J.G., von Keudell A.G., Rodriguez E.K., Smith R.M., Weaver M.J. (2021). Complications and Soft-Tissue Coverage After Complete Articular, Open Tibial Plafond Fractures. J. Orthop. Trauma.

[8] Yeramosu T., Satpathy J., Perdue P.W., Toney C.B., Torbert J.T., Cinats D.J., Patel T.T., Kates S.L. (2022). Risk Factors for Infection and Subsequent Adverse Clinical Results in the Setting of Operatively Treated Pilon Fractures. J. Orthop. Trauma.

[9] Ren T., Ding L., Xue F., He Z., Xiao H. (2015). Risk factors for surgical site infection of pilon fractures. Clin. Sao Paulo Braz..

[10] Kortram K., Bezstarosti H., Metsemakers W.-J., Raschke M.J., Van Lieshout E.M.M., Verhofstad M.H.J. (2017). Risk factors for infectious complications after open fractures; a systematic review and meta-analysis. Int. Orthop..

[11] Wang J., Cambre B.N., Cho D.H., Dugan A.L.M., Panchbhavi V., Wang A.S., Panchbhavi V.K. (2025). Impact of Nicotine Dependence on Postoperative Outcomes in Pilon Fracture Open Reduction and Internal Fixation: A Comparative Cohort Analysis. J. Orthop. Trauma.

[12] Oladeji L.O., Platt B., Crist B.D. (2021). Diabetic Pilon Factures: Are They as Bad as We Think?. J. Orthop. Trauma.

[13] Hebert-Davies J., Kleweno C.P., Nork S.E. (2020). Contemporary Strategies in Pilon Fixation. J. Orthop. Trauma.

[14] Calori G.M., Tagliabue L., Mazza E., de Bellis U., Pierannunzii L., Marelli B.M., Colombo M., Albisetti W. (2010). Tibial pilon fractures: Which method of treatment?. Injury.

[15] Seidelman J.L., Mantyh C.R., Anderson D.J. (2023). Surgical Site Infection Prevention: A Review. JAMA.

[16] Jing C., Ralph J.E., Chang K., Helmkamp J., Krez A., Anastasio A.T., Wu K.A., Cathey J., Bryniarski A., Torrey J. (2025). Risk Factors for Postoperative Infection and Associated Outcomes After Pilon Fracture Fixation: A Propensity-Matched Cohort Study. Foot Ankle Int..

[17] Cuschieri S. (2019). The STROBE guidelines. Saudi J. Anaesth..

[18] Stevens D.L., Bisno A.L., Chambers H.F., Dellinger E.P., Goldstein E.J.C., Gorbach S.L., Hirschmann J.V., Kaplan S.L., Montoya J.G., Wade J.C. (2014). Practice Guidelines for the Diagnosis and Management of Skin and Soft Tissue Infections: 2014 Update by the Infectious Diseases Society of America. Clin. Infect. Dis..

[19] Nicholson J.A., Yapp L.Z., Keating J.F., Simpson A.H.R.W. (2021). Monitoring of fracture healing. Update on current and future imaging modalities to predict union. Injury.

[20] Wittauer M., Burch M.-A., McNally M., Vandendriessche T., Clauss M., Della Rocca G.J., Giannoudis P.V., Metsemakers W.-J., Morgenstern M. (2021). Definition of long-bone nonunion: A scoping review of prospective clinical trials to evaluate current practice. Injury.

[21] Holzer N., Salvo D., Marijnissen A.C.A., Vincken K.L., Ahmad A.C., Serra E., Hoffmeyer P., Stern R., Lübbeke A., Assal M. (2015). Radiographic evaluation of posttraumatic osteoarthritis of the ankle: The Kellgren-Lawrence scale is reliable and correlates with clinical symptoms. Osteoarthr. Cartil..

[22] Cheng H., Chen B.P.-H., Soleas I.M., Ferko N.C., Cameron C.G., Hinoul P. (2017). Prolonged Operative Duration Increases Risk of Surgical Site Infections: A Systematic Review. Surg. Infect. (Larchmt).

[23] Shafiq B., Zhang B., Zhu D., Gupta D.K., Cubberly M., Stepanyan H., Rezzadeh K., Lim P.K., Hacquebord J., Gupta R. (2023). Reducing Complications in Pilon Fracture Surgery: Surgical Time Matters. J. Orthop. Trauma.

[24] Esposito J.G., van der Vliet Q.M.J., Heng M., Potter J., Cronin P.K., Harris M.B., Weaver M.J. (2020). Does Surgical Approach Influence the Risk of Postoperative Infection After Surgical Treatment of Tibial Pilon Fractures?. J. Orthop. Trauma.

[25] Güzel İ., Ulusoy İ., Yılmaz M., Tantekin M.F., Kıvrak A. (2025). Early versus Delayed Plate Fixation in Pilon Fractures. J. Am. Podiatr. Med. Assoc..

[26] Olson J.J., Anand K., von Keudell A., Esposito J.G., Rodriguez E.K., Smith R.M., Weaver M.J. (2021). Judicious Use of Early Fixation of Closed, Complete Articular Pilon Fractures Is Not Associated With an Increased Risk of Deep Infection or Wound Complications. J. Orthop. Trauma.

[27] Kim Y.J., Richard R.D., Scott B.L., Parry J.A. (2023). Acute Fixation Protocol for High-Energy Tibial Pilon Fractures Decreases Time to Fixation and Lowers Operative Costs Without Affecting Wound Complications and Reoperations. J. Orthop. Trauma.

[28] Howard J.L., Agel J., Barei D.P., Benirschke S.K., Nork S.E. (2008). A Prospective Study Evaluating Incision Placement and Wound Healing for Tibial Plafond Fractures. J. Orthop. Trauma.

[29] Molina C.S., Stinner D.J., Fras A.R., Evans J.M. (2015). Course of treatment and rate of successful salvage following the diagnosis of deep infection in patients treated for pilon fractures (AO/OTA: 43). J. Orthop..

[30] Cierny G., Mader J.T., Penninck J.J. (2003). A clinical staging system for adult osteomyelitis. Clin. Orthop. Relat. Res..

[31] Sudduth J.D., Moss J.A., Spitler C.A., Pham V.-L.H., Jones L.C., Brown J.T., Bergin P.F. (2020). Open Fractures: Are We Still Treating the Same Types of Infections?. Surg. Infect. (Larchmt).

[32] Boraiah S., Kemp T.J., Erwteman A., Lucas P.A., Asprinio D.E. (2010). Outcome following open reduction and internal fixation of open pilon fractures. J. Bone Jt. Surg. Am..

[33] Macias-Valcayo A., Aguilera-Correa J.-J., Broncano A., Parron R., Auñon A., Garcia-Cañete J., Blanco A., Esteban J. (2022). Comparative In Vitro Study of Biofilm Formation and Antimicrobial Susceptibility in Gram-Negative Bacilli Isolated from Prosthetic Joint Infections. Microbiol. Spectr..

[34] Hinojosa C.A., Boyer-Duck E., Anaya-Ayala J.E., Nunez-Salgado A., Laparra-Escareno H., Torres-Machorro A., Lizola R. (2016). Impact of the bacteriology of diabetic foot ulcers in limb loss. Wound Repair Regen..

[35] Sen P., Demirdal T., Emir B. (2019). Meta-analysis of risk factors for amputation in diabetic foot infections. Diabetes Metab. Res. Rev..

[36] Gitajn I., Werth P., O’Toole R.V., Joshi M., Jevsevar D., Wise B., Rane A., Horton S., McClure E.A., Ross B. (2022). Microbial Interspecies Associations in Fracture-Related Infection. J. Orthop. Trauma.

